# 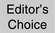 A risk assessment of automated treatment planning and recommendations for clinical deployment

**DOI:** 10.1002/mp.13552

**Published:** 2019-05-06

**Authors:** Kelly Kisling, Jennifer L. Johnson, Hannah Simonds, Lifei Zhang, Anuja Jhingran, Beth M. Beadle, Hester Burger, Monique du Toit, Nanette Joubert, Remigio Makufa, William Shaw, Christoph Trauernicht, Peter Balter, Rebecca M. Howell, Kathleen Schmeler, Laurence Court

**Affiliations:** ^1^ Department of Radiation Physics The University of Texas MD Anderson Cancer Center Houston TX 77030 USA; ^2^ Division of Radiation Oncology Stellenbosch University and Tygerberg Hospital Cape Town 7505 South Africa; ^3^ Division of Radiation Oncology The University of Texas MD Anderson Cancer Center Houston TX 77030 USA; ^4^ Department of Radiation Oncology – Radiation Therapy Stanford University Stanford CA 94305 USA; ^5^ Division of Medical Physics University of Cape Town and Groote Schuur Hospital Cape Town 8000 South Africa; ^6^ Division of Medical Physics Stellenbosch University and Tygerberg Hospital Cape Town 7505 South Africa; ^7^ Department of Medical Physics Gaborone Private Hospital Gaborone Botswana; ^8^ Department of Medical Physics (G68) University of the Free State Bloemfontein 9301 South Africa; ^9^ Department of Gynecologic Oncology and Reproductive Medicine The University of Texas MD Anderson Cancer Center Houston TX 77030 USA

**Keywords:** automated treatment planning, external beam radiation therapy, FMEA, quality assurance, risk analysis

## Abstract

**Purpose:**

To assess the risk of failure of a recently developed automated treatment planning tool, the radiation planning assistant (RPA), and to determine the reduction in these risks with implementation of a quality assurance (QA) program specifically designed for the RPA.

**Methods:**

We used failure mode and effects analysis (FMEA) to assess the risk of the RPA. The steps involved in the workflow of planning a four‐field box treatment of cervical cancer with the RPA were identified. Then, the potential failure modes at each step and their causes were identified and scored according to their likelihood of occurrence, severity, and likelihood of going undetected. Additionally, the impact of the components of the QA program on the detectability of the failure modes was assessed. The QA program was designed to supplement a clinic's standard QA processes and consisted of three components: (a) automatic, independent verification of the results of automated planning; (b) automatic comparison of treatment parameters to expected values; and (c) guided manual checks of the treatment plan. A risk priority number (RPN) was calculated for each potential failure mode with and without use of the QA program.

**Results:**

In the RPA automated treatment planning workflow, we identified 68 potential failure modes with 113 causes. The average RPN was 91 without the QA program and 68 with the QA program (maximum RPNs were 504 and 315, respectively). The reduction in RPN was due to an improvement in the likelihood of detecting failures, resulting in lower detectability scores. The top‐ranked failure modes included incorrect identification of the marked isocenter, inappropriate beam aperture definition, incorrect entry of the prescription into the RPA plan directive, and lack of a comprehensive plan review by the physician.

**Conclusions:**

Using FMEA, we assessed the risks in the clinical deployment of an automated treatment planning workflow and showed that a specialized QA program for the RPA, which included automatic QA techniques, improved the detectability of failures, reducing this risk. However, some residual risks persisted, which were similar to those found in manual treatment planning, and human error remained a major cause of potential failures. Through the risk analysis process, we identified three key aspects of safe deployment of automated planning: (a) user training on potential failure modes; (b) comprehensive manual plan review by physicians and physicists; and (c) automated QA of the treatment plan.

## 1 Introduction

Automation has the potential to improve the consistency and efficiency of radiation treatment planning. Additionally, the automation of treatment planning promises to improve safety by preventing human errors and reducing handoffs between medical staff members, both of which have been shown to be weaknesses in radiotherapy safety.^1^, ^2^, ^3^ While it is generally assumed that automation leads to elimination of the risks associated with human error, it can introduce new or different types of error that are not part of the routine, manual treatment planning process. For example, automated processes may introduce new or added risks from the lack of active participation by a human user who could catch errors that may go undetected by computer algorithms.

As with any other new technology to be implemented into clinical workflow, it is vital to assess the risk introduced by automated treatment planning in each step of the workflow. Furthermore, Task Group 100 (TG‐100) of the American Association of Physicists in Medicine (AAPM) recommends that all new devices undergo a systematic risk analysis.^2^ Failure mode and effects analysis (FMEA) is an established technique for methodically and prospectively identifying the risks involved in a process. This method has been used by several other groups to assess the risks of various processes in radiation oncology practice.^4^, ^5^, ^6^, ^7^, ^8^

Our group recently developed a fully automatic treatment planning tool, the Radiation Planning Assistant (RPA).^9^, ^10^ In addition to developing algorithms to automate treatment planning, we also implemented a quality assurance (QA) program specific to the RPA in order to enhance the safety of automated planning. In the present work, we used FMEA to assess the risk of various failure modes in automated planning for cervical cancer radiotherapy with the RPA. We then assessed the impact of the specialized QA program on the identified risks.

## 2 Materials and methods

### 2.A. Description of the RPA

The workflow of automated planning with the RPA was previously described by Court et al.^9^ Currently, the RPA is being implemented as a remote system with which the user interacts via a web interface. A locally installed system is also possible. The input to the RPA is a plan directive from the physician with patient information, including the treatment site and prescription, and the planning computed tomography (CT) images of the patient in the treatment position. In the RPA's user interface, qualified staff can enter, review, and approve the plan directive and planning CT. Once these are approved, the RPA automatically begins planning.

The RPA is being developed for all treatment sites, starting with cancers of the uterine cervix, head and neck, and breast. Here, we focused on RPA treatment planning for cervical cancer using a four‐field box technique with beam apertures based on bony anatomy. The algorithms and validation results of the RPA for cervical cancer have been previously described.^10^ Briefly, the RPA uses in‐house–developed algorithms that are integrated with the Eclipse treatment planning system (Varian Medical Systems, Palo Alto, CA) via Eclipse's application programming interface. The marked isocenter is automatically localized according to the positions of 3‐point external fiducial markers, and the body contour is automatically segmented. Next, the pelvic bony anatomy is automatically segmented with an autosegmentation tool using deformable registration of multiple atlases.^11^ The pelvic bony anatomy is projected into each beam's‐eye view, and the beam apertures are designed on the basis of anatomical landmarks identified on the projections of the bony anatomy. The treatment beam parameters are then automatically set in the treatment planning system, and the dose is calculated using the Analytical Anisotropic Algorithm. The relative beam weights are optimized to achieve a homogeneous dose distribution within the treated volume.

After planning is complete, the RPA presents the plan as a PDF document for a physician to review. If the physician approves the plan, DICOM‐format treatment plan files are transferred to the user. The expectation is that users will import the files into their own treatment planning systems for review before treatment and will perform their standard pretreatment QA.

### 2.B. FMEA of automated planning

For the FMEA, a team of subject matter experts (three medical physicists and one radiation oncologist) first enumerated the steps in the RPA automated treatment planning workflow for patients with cervical cancer, from CT simulation to plan approval by the physician. Then, for each process step, the group of three physicists identified potential failure modes and possible causes of each failure mode. The process map, failure modes, and scoring were determined assuming a generic clinic that follows the practices outlined in the American College of Radiology accreditation requirements, including physics plan review.^12^ Prior to scoring, the process map and potential failure modes and causes were reviewed by six medical physicists at four centers in South Africa and Botswana that are prospective users of the RPA. These physicists assessed the applicability of the proposed treatment planning workflow and failure modes to their local clinical practice to identify any substantial discrepancies, and none were found.

Next, the original team of three physicists scored the likelihood of occurrence (O), the severity (S), and the likelihood of going undetected (D) for each potential cause of each potential failure mode using the 10‐point scoring system and the values recommended in Table II of the TG‐100 report.^2^ The value for each O, S, and D score was the consensus value as determined by the group. We chose to focus on scoring the causes of failure modes individually in order to better design the QA program to mitigate the variable risks attributable to different causes of a potential failure mode.

A risk priority number (RPN) was calculated by multiplying the O, S, and D scores. For potential failure modes with causes that were specifically related to the failure of an RPA algorithm, we were able to determine the O score quantitatively on the basis of our retrospective testing of the RPA using approximately 500 patient CT scans rather than making an estimate of the percent likelihood of occurrence. For example, a specific algorithm failure that occurred twice during testing would receive an O score of 6, representing an occurrence rate of <0.5%. If a potential failure mode never occurred, we estimated the likelihood of occurrence from our experience and knowledge of the algorithms.

### 2.C. Description of the QA program

To minimize opportunities for error, we implemented a QA program for the RPA that included three types of QA to detect errors in the automatically created treatment plans. We applied this QA program, which is intended to supplement users’ standard QA processes, to all possible steps in the automated planning process. This QA program was initially developed prior to the FMEA based on our experience with the initial retrospective testing and development. The QA program was updated with additional tests based on the results of this FMEA.

The first type of QA applied was an automatic, independent validation of the results of each step, which is a similar concept to the independent dose verification used in radiotherapy. For example, we use two independent methods to automatically detect the marked isocenter based on a 3‐point external fiducial setup. The primary method searches within a band around the body contour for the high‐contrast external fiducials. The secondary method differs in that it searches for high‐contrast objects that constitute a triangle topology. The result of the primary method is used in the treatment plan, and its agreement with the results of the secondary method is verified. If the two methods do not agree, the treatment plan is flagged for human review. Other examples of tasks that have two independent algorithms are segmentation of the body contour and creation of the field apertures. We pushed the treatment plans to Mobius3D (Mobius Medical Systems, Houston, TX) to perform the secondary dose verification. We also automatically verified the patient's orientation and anatomical site (e.g., head vs pelvis) using both the DICOM header information and a simple rigid registration to a full‐body patient CT scan.

The second type of QA was an automatic check of the result against expected values. The expected values may be a range derived from the population of patient plans, such as for field size, or a single value, such as the collimator angle always being equal to 0.

Finally, the third type of QA was a series of specialized manual checks. One such manual check was a set of specific questions in the user interfaces for approving the plan directive and planning CT. For example, we asked the user to verify that the CT scan field‐of‐view was appropriate for the patient. Another manual check was plan documentation that was automatically created and designed to guide the appropriate staff, such as a physicist, through checking the treatment planning parameters.

### 2.D. Assessment of the effect of the QA program

We next assessed the effect of the QA program on the detectability of each failure mode and cause. First, we determined which failure modes could be detected by any of the three types of QA described above. For each type of QA, we estimated how effectively that type of QA could improve the detectability of that failure mode and cause using a three‐tier scale: very effectively, moderately effectively, or somewhat effectively. We used these ratings to adjust the D value (the likelihood of going undetected) for each failure mode by reducing the percentage of undetected failures (from the corresponding score values in Table II from TG‐100). To facilitate the reduction in the D value, we assigned residual percent undetected values of 20%, 50%, and 80% to the very, moderately, and somewhat effective types of QA, respectively, based on the likely effectiveness as determined by group consensus. These reduced values were then converted back to a D score on the 10‐point scale from TG‐100. We then calculated the differences in the RPN of all potential failure modes with and without the QA program.

## 3 Results

**Figure 1:**
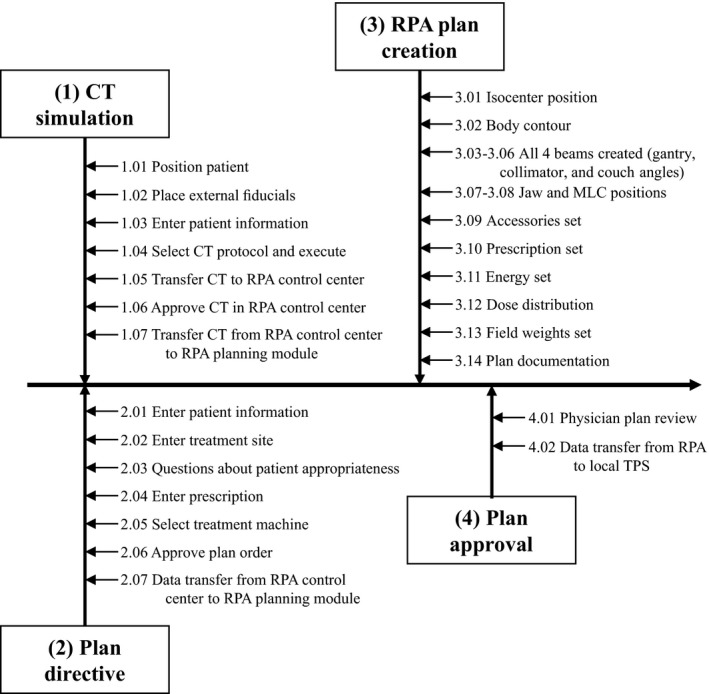
Depiction of the subprocesses and steps involved in automatically planning a four‐field box radiotherapy treatment for cervical cancer with the radiation planning assistant (RPA). Subprocesses 1 and 2 computed tomography (CT simulation and plan directive) involve many manual steps from which errors could propagate downstream. Subprocess 3 (RPA plan creation) is entirely automatic. Abbreviations: MLC, multileaf collimator; TPS, treatment planning system.

**Figure 2:**
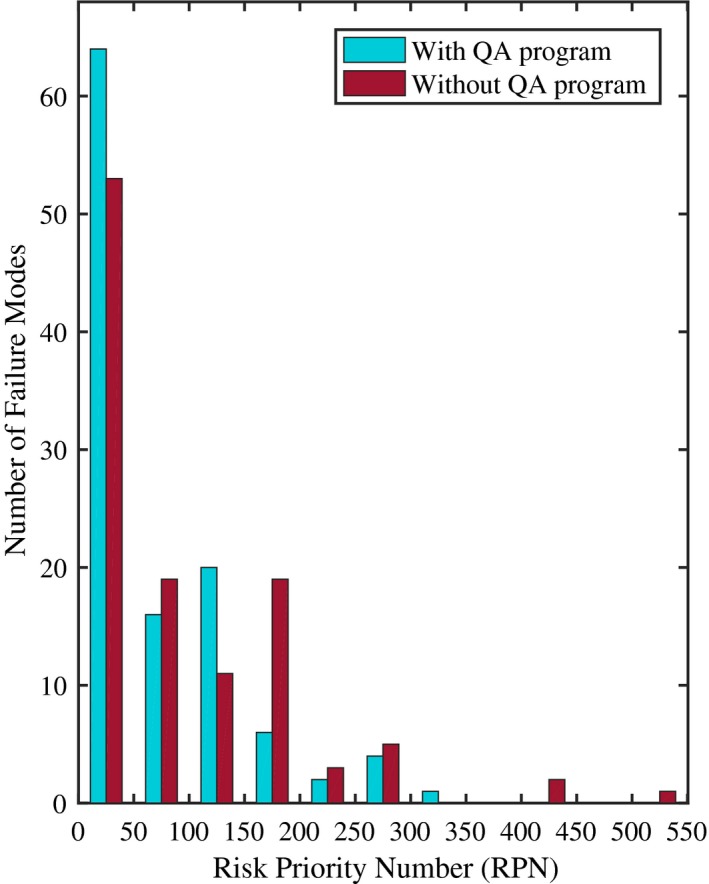
Histogram of the risk priority numbers (RPN) for all potential failure modes identified for automatic planning of a cervical cancer treatment using the radiation planning assistant (RPA) with (blue) and without (red) the quality assurance (QA) program.

**Figure 3:**
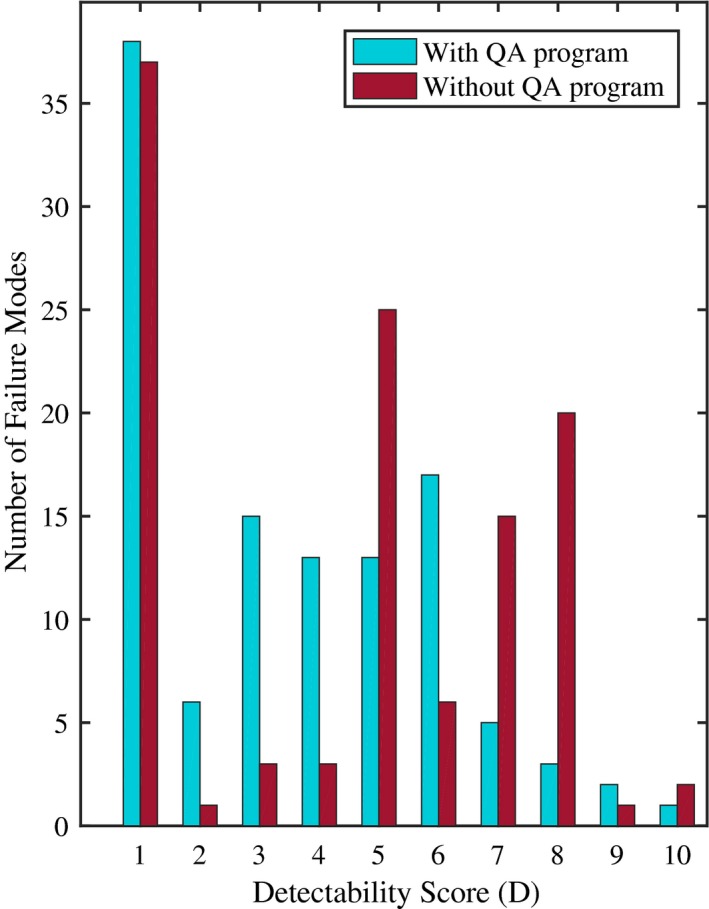
Histogram of the detectability score (D) for all potential failure modes identified for automatic planning of a cervical cancer treatment using the radiation planning assistant (RPA) with (blue) and without (red) the quality assurance (QA) program.

The RPA process, with its four major subprocesses (CT simulation, plan directive, RPA plan creation, and plan approval) and 30 steps, is shown in Fig. 1. Using FMEA, we identified 68 failure modes with 113 potential causes. Of the 113 potential causes, 79 (70%) were subject to at least one type of QA as part of our QA program (not including typical clinical QA practices). Without the QA program, the average RPN was 91, and the maximum RPN was 504. With the QA program, these values were reduced to 68 and 315, respectively. The distribution of the RPNs with and without the QA program for all potential failure modes and causes is shown in Fig. 2, where the overall shift to lower RPN values is apparent. Since the QA program only affected the detectability of failures, we compared the change in the distribution of the D scores with and without the QA program (Fig. 3). The median D score without the QA program was 5.0 and was reduced to 3.0 with the QA program.

Scores for the top ten potential failure modes and associated causes are shown in Tables 1 and 2 for the RPA without the QA program and with the QA program, respectively. Without the QA program, three of the top ten failure modes were related to a failure to correctly identify the marked isocenter based on the external fiducials. A failure in this step could have severe consequences if not detected and is not unique to automated treatment planning. Incorrect definition of the isocenter was also identified as a relatively high‐risk failure mode in an FMEA of pretreatment steps for TomoTherapy by Broggi et al.^5^ The QA program of the RPA includes automatic verification of the isocenter and a guided manual check of the isocenter identification in the plan documentation. With the QA program, only one of the top ten failure modes involved identification of the isocenter. This failure mode was caused by the presence of other external fiducials on the planning CT scan (e.g., wires), which could reduce the effectiveness of the automatic verification QA process.

**Table 1:** The top 10 potential failure modes and their causes in automated planning with the radiation planning assistant (RPA) without the quality assurance (QA) program

#	Major process	Step	Potential failure mode	Potential causes of failure	O	S	D	RPN
1	RPA plan creation	Isocenter position	Incorrectly identified	Other external fiducials	7	9	8	504
2	RPA plan creation	Jaw positions	Inappropriate position	Algorithm error	10	7	6	420
3	RPA plan creation	MLC positions	Inappropriate position	Algorithm error	10	7	6	420
4	Plan approval	Physician plan review	No comprehensive review	Human error	3	10	10	300
5	RPA plan creation	Isocenter position	Incorrectly identified	Algorithm error	4	9	8	288
6	CT simulation	Select CT protocol and execute	Field‐of‐view is too small	Human error	5	8	7	280
7	CT simulation	Select CT protocol and execute	Field‐of‐view is too small	Patient is too large	5	8	7	280
8	Plan directive	Enter prescription	Incorrect (not changed from default)	Human error	4	9	7	252
9	RPA plan creation	Dose distribution	Calculation point is inappropriate	Located in high or low CT number	10	4	6	240
10	RPA plan creation	Isocenter position	Incorrectly identified	External fiducials out of range of scan	3	9	8	216

Abbreviations: O, occurrence score; S, severity score; D, detectability score; RPN, risk priority number; MLC, multileaf collimator; CT, computed tomography.

**Table 2:** The top 10 potential failure modes and their causes in automated planning with the radiation planning assistant (RPA) with the quality assurance (QA) program

#	Major process	Step	Potential failure mode	Potential causes of failure	O	S	D	RPN
1	RPA plan creation	Isocenter position	Incorrectly identified	Other external fiducials	7	9	5	315
2	Plan approval	Physician plan review	No comprehensive review	Human error	3	10	10	300
3	RPA plan creation	Jaw positions	Inappropriate position	Algorithm error	10	7	4	280
4	RPA plan creation	MLC positions	Inappropriate position	Algorithm error	10	7	4	280
5	Plan directive	Enter prescription	Incorrect (not changed from default)	Human error	4	9	7	252
6	CT simulation	Select CT protocol and execute	Field‐of‐view is too small	Human error	5	8	6	240
7	CT simulation	Select CT protocol and execute	Field‐of‐view is too small	Patient is too large	5	8	6	240
8	Plan directive	Questions about patient appropriateness	Completed incorrectly	Human error	4	9	5	180
9	Plan directive	Approve plan directive	Approved by staff without correct rights	Shared login/Incorrect rights	4	9	5	180
10	CT simulation	Position patient	Inappropriate positioning	Human error	6	4	7	168

Abbreviations: O, occurrence score; S, severity score; D, detectability score; RPN, risk priority number; MLC, multileaf collimator; CT, computed tomography.

Other important potential failure modes, with and without the QA program, were the definition of the beam apertures (jaws and multileaf collimator). We implemented automatic QA verification to detect gross errors in beam aperture definition. However, it remains vital that the physician review the appropriateness of the beam apertures, regardless of whether the beam apertures are determined automatically by computer algorithms or drawn manually by a dosimetrist or resident. In fact, a potential failure mode identified by both our FMEA and that of other groups was failure of the physician review of plan quality.^2^, ^6^ While physician review is standard clinical practice, and such a failure is unlikely, it would be very difficult to detect and could have severe consequences if the plan quality is inadequate. We have previously shown that nonspecialists are unlikely to reliably identify these errors, even when presented with examples of correct beam apertures as a reference.^9^

We specifically investigated the 15 higher risk failure modes — those for which the RPN was greater than or equal to 200. Of these, 13 (87%) were subject to at least one type of QA technique. The number of these higher risk failure modes was reduced from 15 to 7 when the QA program was implemented, with an average reduction in RPN score of 93 points (range, 40–189). Physician review of the plan was one of the two higher risk failure modes that could not be mitigated by a specialized QA technique (beyond standard clinical practice).

The other top failure mode that was not subject to a specialized QA technique was an incorrectly entered prescription in the plan directive workspace, specifically cases in which the physician intended to change the prescription from the default or typical value. While such a scenario is unlikely, this failure would be more difficult to catch later in a manual check, since the intended prescription would not be the expected, typical value for that patient type. Automation does mitigate many potential transcription errors (such as those that occur between the plan directive and the treatment planning system). However, the initial entry into the system must be as the planning physician intends, regardless of whether manual or automated treatment planning is used. In fact, incorrect prescription has been identified as a potentially severe failure mode by other groups studying manual treatment planning.^5^, ^6^

We also investigated the 22 failure modes and associated causes with high‐severity scores (S ≥ 9). Table 3 lists all failure modes and causes with S scores of 9 or higher, including their overall scores with the specialized QA program. Here, we found that 14 (64%) failure modes were subject to at least one type of QA. On average, the RPN of the high‐severity failure modes was reduced from 146 to 113 with the QA program implemented. The maximum RPN for these was reduced from 504 to 315. Most of these failure modes were unlikely to occur; 20 (91%) had O scores of 4 or less. With one exception, the high‐severity potential failure modes that were not subject to any specialized QA test were caused by human error, such as incorrectly entering the prescription in the plan directive.

**Table 3:** Potential automated treatment planning failure modes and associated causes with severity (S) scores of 9 or higher. Scores shown are for the radiation planning assistant (RPA) with the quality assurance (QA) program implemented

Major process	Step	Potential failure mode	Potential causes of failure	O	S	D	RPN
CT simulation	Enter patient information	Incorrect name or ID entered	Human error	1	10	9	90
Plan directive	Enter patient information	Incorrect name or ID entered	Human error	2	10	1	20
RPA plan creation	Prescription set	Does not match the plan directive	Algorithm error	1	10	5	50
RPA plan creation	Prescription set	Incorrect normalization	Algorithm error	1	10	5	50
RPA plan creation	Dose distribution	Calculation point not at isocenter	Algorithm error	1	10	5	50
RPA plan creation	Plan documentation	Data corrupted	Algorithm error	3	10	4	120
Plan approval	Physician plan review	No comprehensive review	Human error	3	10	10	300
Plan approval	Data transfer from RPA to local TPS	Data corrupted	Network error	2	10	3	60
CT simulation	Position patient	Incorrect orientation	Human error	3	9	2	54
CT simulation	Position patient	Incorrect orientation	Standard technique varies from RPA protocol	6	9	1	54
CT simulation	Position patient	Incorrect orientation	Intentional nonstandard technique	4	9	2	72
Plan directive	Questions about patient appropriateness	Completed incorrectly	Human error	2	9	7	126
Plan directive	Questions about patient appropriateness	Completed incorrectly	Human error	4	9	5	180
Plan directive	Enter prescription	Incorrect (not changed from default)	Human error	4	9	7	252
Plan directive	Enter prescription	Incorrect (changed from default)	Human error	3	9	6	162
Plan directive	Approve plan order	Approved by person without correct rights	Shared login/incorrect rights	4	9	5	180
RPA plan creation	Isocenter position	Incorrectly identified	Other external fiducials	7	9	5	315
RPA plan creation	Isocenter position	Incorrectly identified	Fiducials out of range of CT	3	9	4	108
RPA plan creation	Isocenter position	Incorrectly identified	Algorithm error	4	9	4	144
RPA plan creation	All 4 beams created	Not created at the isocenter	Algorithm error before aperture generation	1	9	6	54
RPA plan creation	All 4 beams created	Not created at the isocenter	Algorithm error after aperture generation	1	9	2	18
RPA plan creation	MLC positions	MLC missing from plan	Algorithm error	1	9	2	18

Potential failures in the “Plan directive” step “Questions about patient appropriateness” were scored by considering two separate scenarios: (a) when the result does not affect the results of automated planning, but still poses a risk (such as prior irradiation); and (b) when the result would technically affect the result of automated planning (such as the presence of an artificial hip, which may cause errors in contouring the bony anatomy).

Abbreviations: O, occurrence score; D, detectability score; RPN, risk priority number; CT, computed tomography; TPS, treatment planning system; MLC, multileaf collimator.

## 4 Discussion

Following the recommendation of TG‐100 to perform a risk analysis of new technologies to be implemented in clinical practice, we assessed the risk of our recently developed automated treatment planning tool, the RPA. To our knowledge, this is the first published work assessing the risk of automated treatment planning using FMEA. Additionally, we determined the effect of the specialized QA program on the RPA's failure risk. This work has enabled us to systematically and prospectively identify the highest risk steps involved in our automated treatment planning workflow and aided us in developing a QA program specific to the RPA.

While the FMEA presented here is specific to the workflow of the RPA, the lessons gained can be applied broadly to implementations of automated planning. The current analysis has shown us that many of the highest risk steps, both with and without the QA program (as shown in Tables 1 and 2), are similar to what might be expected in a manual treatment planning process, including correct identification of the marked isocenter, use of appropriate beam apertures, and use of the correct prescription. However, while the resulting failures may be the same, the causes of these failures may be different in automated planning, in which an algorithm, rather than a human planner, may fail to perform adequately. We have found that many of the highest risk errors in our automated planning workflow were caused by human error. Such errors in an automated plan can be readily detected by the physicist or physician who reviews the plan, as in standard clinical practice. The results of this study emphasize the importance of these plan reviews prior to patient treatment, regardless of whether the plan was generated automatically or using standard manual techniques. In fact, with the advent of automated treatment planning, we need to ensure that we do not develop an overreliance on automation and forego the usual attention to detail in the manual review of treatment plans. In general, automation can improve the safety and consistency of many steps of treatment planning, especially for more objective tasks such as setting prescriptions and creating the beams at the correct isocenter. However, for more subjective tasks, such as designing treatment beams or contouring, the automation techniques and algorithms, like a human planner, have a certain level of skill, so their results should always be scrutinized by qualified staff.

Using automated QA techniques for plan review has been proposed by several groups.^13^, ^14^, ^15^, ^16^ Here, we incorporated such checks for the consistency and reasonability of the treatment planning parameters as part of our QA program. Additionally, we included automatic verification of more subjective tasks, such as designing beam apertures. These automatic checks add additional risk mitigation without additional workload on staff, which is especially important for resource‐constrained settings. However, our QA program only addresses potential failure modes in treatment planning and does not address failures that may occur as plans are transferred to the local treatment planning system or record‐and‐verify system, although we have developed software to check the integrity of this data transfer.

In addition, our study revealed that automation of treatment planning and QA does not completely remove the risk of human error. For example, the effect of an incorrectly entered prescription is still a high‐risk point that can only be detected by a diligent human review. To address this, the third component of our QA program, the specialized manual checks, was designed to draw the reviewer's attention to these important components of the treatment plan. However, there are still potential failures that are not covered by any of our QA program, including the specialized manual checks. Many of these potential failures could be caught by standard QA steps, such as the typical physics and physician plan reviews which could detect if there was a discrepancy of the prescription or an abnormality in the plan. To reinforce the importance of the manual checks as we clinically implement automated planning, training of the users of the RPA will be vital to overcoming the residual higher risk failure modes. The results of this FMEA will inform the training by educating the users on what are the highest risk potential failure modes that should be included in their manual checks.

Our analysis of approximately 500 test cases was able to quantify some of the values for the likelihood of occurrence of potential failure modes caused by algorithm error. However, most of the failure modes we identified prospectively through the FMEA did not materialize in this testing. Therefore, the O scores were estimated for these potential failure modes on the basis of the FMEA team's experience and knowledge of the RPA algorithms. In order to further quantify and improve the reliability of the FMEA results, more extensive testing will be necessary in the course of pre‐ and early implementation. Therefore, the results of this FMEA will be a living document that will be updated as more testing occurs. This is similar to techniques that incorporate data from incident‐learning systems to validate and improve upon FMEA findings. We intend to conduct regular reviews and, if necessary, update the FMEA throughout the testing, clinical implementation, and use of the RPA. By continuing to monitor the RPA's performance, the occurrence of failures, and the ability of the QA program to detect failures, we will collect quantitative data that will improve the validity of the FMEA.

Additionally, before implementing automated planning for other treatment sites or techniques using the RPA, we will reassess the process map and failure modes. For example, in planning volumetric modulated arc therapy for head and neck cancers, contouring is a vital step that has been identified by other groups as a high‐risk potential failure mode.^2^, ^5^, ^7^ Therefore, this FMEA will be updated to consider the risk of failures in automatic contouring, and QA techniques will be added to reduce the risks of these failures.

The FMEA is by nature subjective; it incorporates the experience and bias of the team performing it. Thus, the results of this analysis may not necessarily represent the true risk of the system. Still, FMEA is a valuable tool for prospectively identifying potential failure modes and risks, which can help to design QA programs to mitigate the identified risks. Given this limitation, continuous monitoring of the performance of automated planning tools is vital to the safe use of automated treatment planning. Moreover, because the RPA is intended for global use in clinics that may have various levels of resources and follow different practice guidelines, it is important to assess how any deviations from the procedures and workflows assumed to be in place for this FMEA will affect its evaluations of risk. As part of initial clinical implementation, the results of this FMEA will be reassessed with multidisciplinary teams from each clinic to ensure the results are reflective of each clinics particular risk profile.

Finally, on the basis of the results of this FMEA and the experience we gained through this process, we identified the following three key components of the safe deployment of automated treatment planning:

1. **Training.** Training should educate the end users of automated planning systems about the potential failure modes, the impact of these failures on patients, and the need for careful manual review of the plans to prevent these failures.
2. **Manual plan checks.** Physician review of the plans (and contours, where necessary) and physics checks are essential components of automated treatment planning.
3. **Automated QA.** It is important to not only automate the planning but also to include automated QA steps, as these can substantially mitigate the risks of automated planning.

## 5 Conclusions

We carried out an FMEA to assess the risk involved in the clinical deployment of automated treatment planning. We determined that while automated QA reduces the risks of automated planning, effective training and manual plan checks by radiation oncologists and physicists remain extremely important parts of the deployment process.

## Conflicts of Interest

This work was partially supported by Varian Medical Systems and Mobius Medical Systems.
